# Magnetic Behaviour of Mn_12_-Stearate Single-Molecule Magnets Immobilized on the Surface of 300 nm Spherical Silica Nanoparticles

**DOI:** 10.3390/ma13112624

**Published:** 2020-06-09

**Authors:** Magdalena Laskowska, Oleksandr Pastukh, Piotr Konieczny, Mateusz Dulski, Marcin Zalsiński, Lukasz Laskowski

**Affiliations:** 1 Institute of Nuclear Physics Polish Academy of Sciences, PL-31342 Krakow, Poland; magdalena.laskowska@ifj.edu.pl (M.L.); piotr.konieczny@ifj.edu.pl (P.K.); lukasz.laskowski@ifj.edu.pl (L.L.); 2 Silesian Center for Education and Interdisciplinary Research, Institute of Materials Science, Institute of Materials Science, Faculty of Computer Science and Materials Science, University of Silesia, ul. 75 Pułku Piechoty 1A, 41-500 Chorzów, Poland; mateusz.dulski@smcebi.edu.pl; 3 Institute of Computational Intelligence, Czestochowa University of Technology, 42-200 Czestochowa, Poland; marcin.zalasinski@iisi.pcz.pl

**Keywords:** single-molecule magnet, Mn_12_, surface functionalization, separation, SQUID

## Abstract

The magnetic behaviour of Mn${}_{12}$-stearate single-molecule magnets (SMMs) ([${\mathrm{Mn}}_{12}O_{12}$${\left({\mathrm{CH}}_{3}{\left({\mathrm{CH}}_{2}\right)}_{16}{\mathrm{CO}}_{2}\right)}_{16}]\cdot 2{\mathrm{CH}}_{3}\mathrm{COOH}\cdot 4H_{2}O$) on the surface of 300 nm spherical silica nanoparticles were investigated. The SMMs were bonded at the silica surface with the assumed number of anchoring points, which influenced on their degree of freedom and distribution. In order to check the properties of Mn${}_{12}$-stearate molecules separated on the silica surface, and check their interactions, the samples containing four different concentration of spacers per single anchoring unit and variously bonded Mn${}_{12}$-stearate particles were prepared. The materials have been examined using Raman spectroscopy, transmission electron microscopy, and SQUID magnetometry. The results of magnetic measurements showed a correlation between the way of single-molecule magnets immobilization onto the silica spheres and the magnetic properties of the obtained hybrid materials.

## 1. Introduction

Since the discovery of single-molecule magnets in 1991 [1,2], the prototype of which is the Mn${}_{12}$ cluster [3], scientists are looking for a way to use them in nanoelectronics applications. One of the main challenges on the path to creating electronic devices using single-molecule magnets (SMMs) is the ability to manipulate individual molecules while maintaining their magnetic properties. The use of magnetic molecules in applications like data-storage or spintronic devices requires the ability of precise placing the molecules on the surface, separating them and addressing their positions [4]. Therefore, scientists are looking for Mn${}_{12}$ derivatives and other magnetic molecules suitable to deposition on the surface. Finding a material that is easily soluble and durable in atmospheric conditions is a great challenge. Another difficulty is the choice of research techniques that allow observation of individual molecules and the measurement of their properties. Scientists have put a lot of effort in recent years to overcome these difficulties and describe the properties of various magnetic molecules deposited on surfaces [5,6,7,8]. However, the procedures for doing this are very demanding and require a sophisticated method for confirming the structure of material [9]. Moreover, very often, the anchored SMMs lose its magnetic properties after separation and deposition on the surface [10].

Our team has designed the synthesis route allowing for the separation of the stearate derivative of Mn${}_{12}$ clusters [11] ([${\mathrm{Mn}}_{12}O_{12}{\left({\mathrm{CH}}_{3}{\left({\mathrm{CH}}_{2}\right)}_{16}{\mathrm{CO}}_{2}\right)}_{16}]\cdot 2{\mathrm{CH}}_{3}\mathrm{COOH}\cdot 4H_{2}O$ hereafter called Mn${}_{12}$-st – the structural formula clarified below) onto the surface of spherical silica [12,13]. This robust procedure enables not only for separating the magnetic molecules but also allows to control the distance between them in a statistical way. Such a possibility opens wide perspectives for the investigation of the magnetic properties and intermolecular interactions of the separated SMMs, as well as interactions between SMMs and the surface of the substrate.

**Figure 1:**
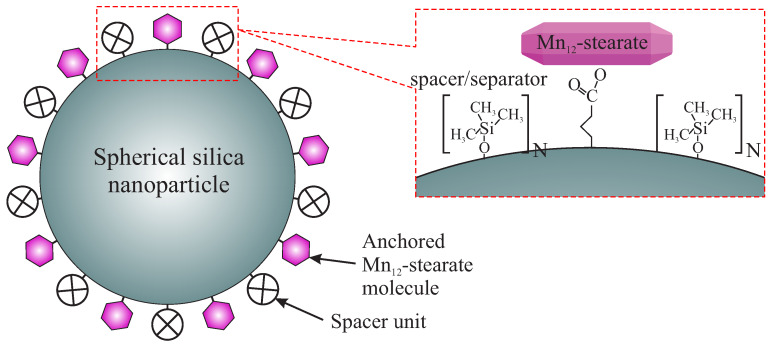
A schematic illustration of the investigated material. N denotes the number of spacer units, separating anchored Mn${}_{12}$-st single-molecule magnets.

The motivation of the present work is to investigate the effect of the concentration of spacer units on the silica surface on the magnetic properties of this material (being the nanocomposite consisted of Mn${}_{12}$-st and spherical silica substrate). To demonstrate how the concentration of spacer units affects the static and dynamic magnetic properties, we prepared four samples with the various distribution of Mn${}_{12}$-st SMMs and different way of its immobilization (implying various degree of freedom) at the silica surface (we prepared four samples: three of them posses highest concentration of the SMMs, but various number of their anchoring points, and one has significantly lower concentration of magnetic molecules—samples are listed and described in the Section 2.1). The assumed concentration of anchoring points (influencing on the degree of freedom of SMMs and their distribution for some cases) is preserved thanks to the use of spacer units [14]. The structure of the materials is presented in Figure 1.

## 2. Materials and Methods

### 2.1. Samples Synthesis

The Mn${}_{12}$/silica spheres hybrid material was prepared according to a procedure described in details in our earlier work [13].

In this place we would like to remark, that we assume total substitution of acetate groups at the Mn${}_{12}$ core by stearic acid units during the preparation of the Mn${}_{12}$-st SMMs. In the source publication [11] authors claim, that only 11 of 16 total acetate units are substituted, and the structural formula of Mn${}_{12}$-st SMMs is as follow: [${\mathrm{Mn}}_{12}O_{12}{\left({\mathrm{CH}}_{3}{\left({\mathrm{CH}}_{2}\right)}_{16}{\mathrm{CO}}_{2}\right)}_{11}{\left({\mathrm{CH}}_{3}{\mathrm{CO}}_{2}\right)}_{5}]\cdot 2{\mathrm{CH}}_{3}\mathrm{COOH}\cdot 4H_{2}O$. We carried out the reaction with elongated reaction time to 24 h under the protective atmosphere and with the use of finely grounded Mn${}_{12}$-ac. Elemental analysis of the resulting powder pointed out the total substitution of acetate groups by stearic acid units. For this reason, we assume the following structural formula of Mn${}_{12}$-st: [${\mathrm{Mn}}_{12}O_{12}{\left({\mathrm{CH}}_{3}{\left({\mathrm{CH}}_{2}\right)}_{16}{\mathrm{CO}}_{2}\right)}_{16}]\cdot 2{\mathrm{CH}}_{3}\mathrm{COOH}\cdot 4H_{2}O$.

**Figure 2:**
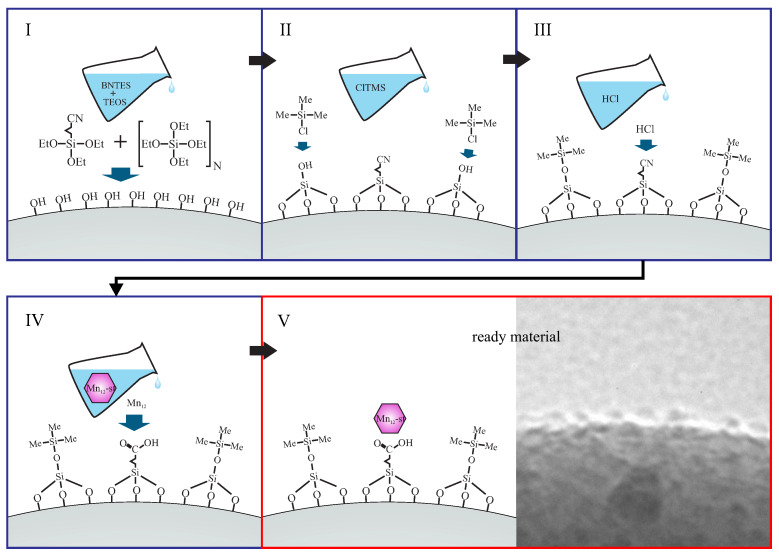
A schematic illustration of the synthesis procedure of investigated material: nanocomposite contained of spherical silica and Mn${}_{12}$-st molecules onto its surface with the assumed statistical distances. Denotations: TEOS—tetraethyl orthosilicate, BNTES—butyronitriletriethoxysilane, ClTMS—chlorotrimethylsilane, Me—methyl groups, Et—ethyl units.(For details see Reference [13]).

A very concise presentation of the synthesis route for functionalization of spherical silica by Mn${}_{12}$-st can be seen in Figure 2. The statistical distances between Mn${}_{12}$-st molecules can be tuned by the variation of the proportion between precursors of spacer units (tetraethyl orthosilicate) and anchoring groups (butyronitriletriethoxysilane), which is defined by the N number in Figure 2.

As a substrate we selected the spherical silica with the diameter of 300 nm as possessing a relatively large specific surface area of 12 m${}^{2}$/g. This materials was prepared according to the optimized Stöber protocol [15]. To investigate the magnetic properties of the composite materials presented here, we fabricated the materials containing four various concentrations of anchoring units at the surface, defined by the number of spacer units per single anchor: one, three, six and nine. The concentration of anchoring groups determines the degree of freedom of bonded Mn${}_{12}$-st, and below some critical content, also the concentration of magnetic molecules. Samples were named SilS-Mn${}_{12}$N1, SilS-Mn${}_{12}$N3, SilS-Mn${}_{12}$N6 and SilS-Mn${}_{12}$N9 respectively, where number just after N denotes the number of separator groups per single SMM. The sample with a higher concentration of anchoring groups is SilS-Mn${}_{12}$N1, while the lowest concentration of anchors can be found at the SilS-Mn${}_{12}$N9 sample. Individual samples can be characterized as follow:

- SilS-Mn${}_{12}$N1: sample with a highest possible concentration of Mn${}_{12}$-st at the surface and the most rigidly anchored molecules (multiple bonds between silica surface and the SMMs);
- SilS-Mn${}_{12}$N3: sample with a highest possible concentration of Mn${}_{12}$-st at the surface, rigidly anchored molecules but lower number of bonds between silica surface and the SMMs, than for the previously listed sample;
- SilS-Mn${}_{12}$N6: sample with a highest possible concentration of Mn${}_{12}$-st at the surface bonded via single bonds—free-floating molecules of Mn${}_{12}$;
- SilS-Mn${}_{12}$N9: sample with a significantly lower concentration of Mn${}_{12}$-st at the surface bonded via single bonds—free-floating molecules of Mn${}_{12}$.

### 2.2. Characterization Methods

The presence of magnetic molecules on the surface of silica spheres and the correlation between concentration and distribution of molecules on the surface were confirmed by using Transmission Electron Microscopy (TEM). Imaging was carried out by applying the FEI Tecnai G2 20 X-TWIN electron microscope, equipped with emission source LaB6, Charged Coupled Device (CCD) camera FEI Eagle 2 K.

The Raman spectra of as prepared samples were recorded with the use of the WITec Confocal Raman Microscope (CRM) alpha 300R which is equipped with an air-cooled solid-state laser (30 mW output power) and a CCD camera. Raman scattering measurements have been performed using 532 nm laser radiation. The laser radiation power at the sample was 5 mW. All recorded spectra were manipulated by peak fitting analysis, baseline correction and cosmic ray removal in the GRAMS software package. Additionally, the spectra were normalized to the silica bands to confirm the impact of various concentrations of Mn${}_{12}$-st groups.

Magnetic properties of Mn${}_{12}$/silica spheres hybrid material were collected using the Quantum Design Magnetic Property Measurement System (MPMS) magnetometer for the samples in the form of powder. Isothermal magnetization *M(H)* was measured at 2.0 K in the magnetic field range −70 kOe to 70 kOe for all samples. DC magnetic susceptibility was measured in zero-field cooling (ZFC) and field cooling (FC) modes, in the temperature range of 2.0 K–20 K under the external magnetic field *H* = 100 Oe. The magnetic relaxations were analyzed by time-dependent magnetization measurements in the temperature range 2.0 K–3.0 K. For that purpose, the magnetic field of 50 kOe was applied and stabilized for five minutes. After the magnetization had become stable, the field was switched off, and the dependence of magnetization over time was measured. The diamagnetic contribution from the silica substrate was taken into account. For such a purpose analogous magnetic measurements (using the analogous conditions and parameters) were performed for the reference sample, pure spherical silica without Mn${}_{12}$ functionalities. Then obtained data for every measurement were subtracted from corresponding magnetic data of investigated samples.

## 3. Results and Discussion

### 3.1. Structural Investigations

**Figure 3:**
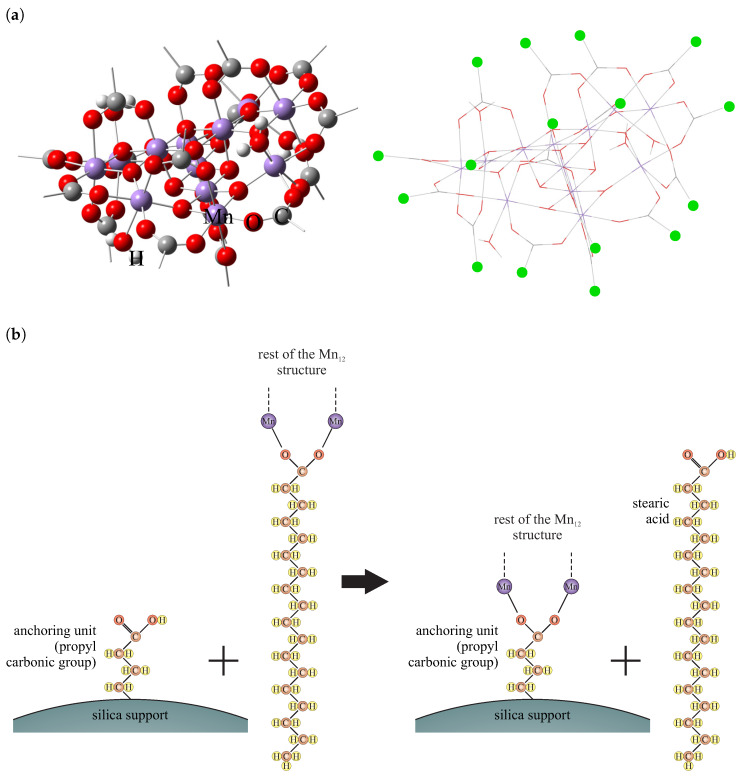
A model of the Mn${}_{12}$ core with carboxylic acid units (-COO) (left side presented as a balls and bonds, right side as sticks for better visualization) (**a**). Alkane chains are omitted for clarity. At the stick diagram: green dots are a points of the attaching of the alkane chains and anchoring units from silica. At the figure (**b**) the way of Mn${}_{12}$ attaching to the surface of the pre-functionalized silica.

The evidence of Mn${}_{12}$-st molecules presence on the silica surface and their distribution was done by TEM imaging supported by differential pulse anodic stripping voltammetry (DPASV). Results were analyzed in details in our previous work [13]. In this work, we kept the same samples nomenclature for clarity. Here, we present only the most important details, revealed by the microscopy: the number of the spacer units affect not only the distribution of the Mn${}_{12}$-st molecules but also the way of its bonding. This fact is important for further interpretation of the results of the magnetic measurements. To understand this fact properly let us consider the structure of Mn${}_{12}$-based molecules and the way of their attaching to the pre-functionalized silica surface. Figure 3a shows the structure of the Mn${}_{12}$ core with COO units. Alkane chains are omitted for clarity (for the case of Mn${}_{12}$-st it would be ${\left({\mathrm{CH}}_{2}\right)}_{16}-{\mathrm{CH}}_{3}$ chains). The way of attaching to the silica is presented in Figure 3b.

**Figure 4:**
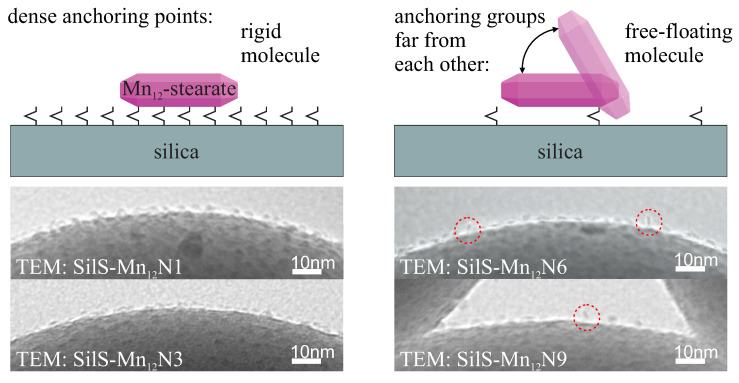
Visualization of the way of Mn${}_{12}$ single-molecule magnets immobilization onto a silica surface, depending on the density of anchoring units (upper side), along with transmission electron microscopy (TEM) images of samples (bottom part).

It can be clearly seen, that the Mn${}_{12}$-based molecule posses 16 attaching points: eight at the circumference, four at the upper side and next four at the bottom. Assuming the umbrella-like configuration of the anchored Mn${}_{12}$[12], the SMMs can be attached through 1–4 points. One can imagine, that the number of bonds between the surface of the silica and SMMs depends on the density of anchoring units at the silica surface—when anchors are densely placed, the Mn${}_{12}$ can be bonded via multiple points (two, three or even four). In this case, SMM is relatively rigid. For the anchoring units placed relatively far from each other, the magnetic molecule can be bonded by a single point and can be easily spatially reoriented. This situation was depicted in Figure 4, along with TEM images confirming our theory.

Closer inspection to the bottom part of Figure 4 confirms our assumption. As we described earlier [13], the concentration of SMMs do not change with increasing of the spacer units up to the proportion of 6 spacers per single anchoring unit (sample SilS-Mn${}_{12}$N6). This sample (SilS-Mn${}_{12}$N6) is very important however because such a concentration of attaching groups still keeps the highest possible concentration of the Mn${}_{12}$, but molecules on the surface exhibit greater mobility, as if they could rotate in space above the surface being attached only at one point. For the higher amount of spacer units (sample SilS-Mn${}_{12}$N9), the concentration of the magnetic molecules significantly decreases (molecules are free-floating, similarly to SilS-Mn${}_{12}$N6). For further details, see our earlier work [13] (see also the Supplementary Information of this article).

Thus, we can divide investigated samples into two groups—materials with decreasing concentration of magnetic molecules on the silica surface (SilS-Mn${}_{12}$N6 and SilS-Mn${}_{12}$N9) with grater mobility of molecules, probably bounded via only one anchoring group and the second group of materials with the highest possible concentration of magnetic molecules on the silica surface (the same as for SilS-Mn${}_{12}$N6), but rigidly attached (SilS-Mn${}_{12}$N1 and SilS-Mn${}_{12}$N3).

**Figure 5:**
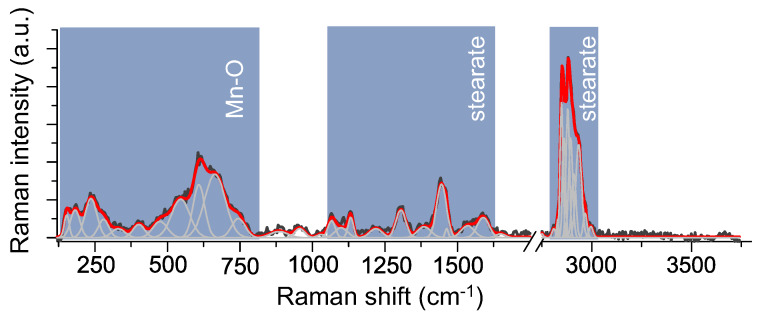
Raman spectra of reference sample: bulk Mn${}_{12}$-st.

The supported structural characterization of molecular magnets was done on the basis of the Raman spectroscopy. To correctly analyze the two-composite system, firstly, it is crucial to analyze and interpret more precisely the reference bulk Mn${}_{12}$-st (Figure 5). The Raman spectrum of bulk molecular magnets may be divided into three spectral regions: (i) 200–770 cm${}^{-1}$ determined by stretching and deformational vibration of Mn-O bonds, (ii) 1100–1800 cm${}^{-1}$ assigned to deformational modes of methyl and methylene groups of the stearate and stretching modes of coordinated carboxyl groups, (iii) 2800–3100 cm${}^{-1}$ linked to the symmetric and asymmetric stretching vibration of methyl (CH${}_{3}$) and methylene (CH${}_{2}$) groups in alkyl configuration of stearate [16,17,18].

**Figure 6:**
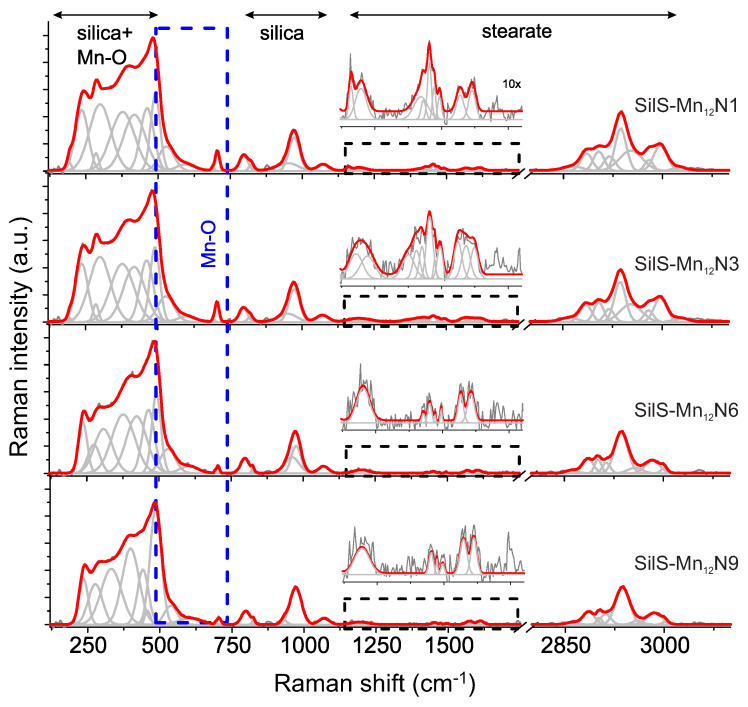
Raman spectra of investigated composite samples containing various concentration of Mn${}_{12}$-st molecules attached at the surface of spherical silica.

In turn, the analysis of Raman spectra of the surface-functionalized silica-based systems (see: Figure 6) turned out to be problematic. It is because the strongest bands originated from Mn${}_{12}$-st become very low in the intensity in comparison to bands typically ascribed to the silica bands. It is quite problematic in the low-frequency region, especially considering bands originated from Mn-O modes which generally overlap with the silica bands. It seems that only band located at 704 cm${}^{-1}$ can be analyzed whereby its intensity becomes still relatively low. A similar scheme was found for all analyzed composites. It is also observed that the intensity of Mn-O band seems to different, considering systems with variable concentration of anchoring units, that is, it is similar for two highest content of rigidly anchored molecular magnets (samples SilS-Mn${}_{12}$N1 and SilS-Mn${}_{12}$N3) and slightly lower for samples SilS-Mn${}_{12}$N6 and SilS-Mn${}_{12}$N9. However, the more precise analysis of the number of molecular magnets anchored to the silica seems to be difficult and burdened of some uncertainty. Thus, crucial is to look more in detail at another spectral range.

The 1100–1800 cm${}^{-1}$ region is less affected by silica carrier and in the case of Mn${}_{12}$-st is mainly characterized by stearate modes, that is, methyl, methylene and carboxyl groups [16]. Unfortunately, similar like previously the low intensity of these bands resulted from very low contribution of the Mn${}_{12}$-st in the composite material, even for high doping rate system. Despite some limitations, the Raman spectrum illustrated in some magnification revealed a band arrangement typically for bulk material. It turned out that the band arrangement as for the number, as well as intensity for samples SilS-Mn${}_{12}$N1 and SilS-Mn${}_{12}$N3, are very similar. These findings seem to be in full accordance with our assumptions: sample posses the same concentration of the SMMs, the difference lays in the number of attaching points. A gradual decrease of the anchoring units led to a significant lowering of the band’s intensity and their number for the other two samples (SilS-Mn${}_{12}$N6 and SilS-Mn${}_{12}$N9). This observation for SilS-Mn${}_{12}$N6 is very surprising since according to our earlier findings, this sample should contain the same number of the magnetic molecules, like previously mentioned ones [13]. Furthermore, this fact is well-confirmed by the microscopic observation, but at this moment, we are not able to clarify it. In turn, the spectroscopic effect found for the SilS-Mn${}_{12}$N9 is in accordance with our assumption because of the very low concentration of magnetic units.

The two relatively low intense bands centred around 1612 and 1576 cm${}^{-1}$ derive from the carboxyl C-O stretching modes and correlate with the formation of Mn${}_{12}$ complex with the ligated carboxylates in bidentate stearic acid. It is difficult to say anything about changes of their intensities with modification of the doping rate, but their presence in all analyzed samples proofed correctness of the synthesis procedure.

Some interesting findings might be observed in the case of the bands from the region located at 2800–3100 cm${}^{-1}$ corresponding to the stretching vibration of methyl and methylene groups in alkyl configuration of stearate [16,17,18]. The analysis of the bands of this region seems to be the most reliable because of their high intensity, especially in relation to other stearate bands. It is also worth noting here that the signal was previously calibrated to the most, intense silica bands. According to this assumption, it seems that the intensity of CH${}_{x}$ (x = 2, 3) bands insignificantly lower with the intensity when the number of anchoring molecular magnets units decreasing. However, this change seems to be gentle, and it is difficult to unambiguously estimate the correctness of this observation.

As a result, the Raman analysis seems to be not enough for reliable detection of changes in the concentration of Mn${}_{12}$-st in the composite. We can only confirm the presence of SMMs at the silica surface and its correct configuration. Hence, our earlier investigations (TEM and differential pulse anodic stripping voltammetry (DPASV) ) [13] were taken into consideration to follow the variation in the Mn${}_{12}$ concentration.

### 3.2. Magnetic Studies

**Figure 7:**
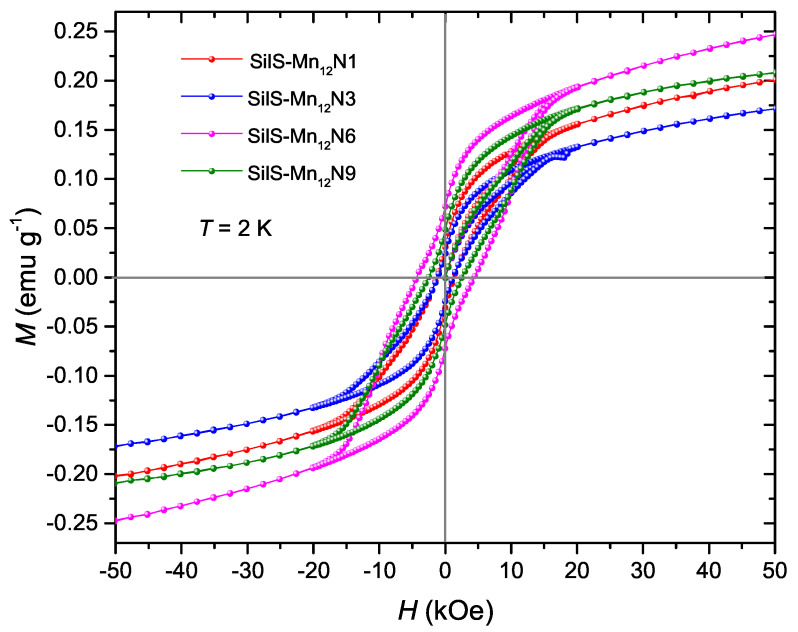
Isothermal magnetization for the samples with different spacer to functional unit ratio at *T* = 2.0 K.

In order to compare the magnetic properties of the Mn${}_{12}$-st molecules deposited on the surface with different concentration of spacers per single anchoring unit, a series of static and dynamic magnetic measurements were performed. Figure 7 shows the dependence of magnetization on the applied field, measured at 2.0 K. For all samples, the measurements reveal visible hysteresis loops confirming the preservation of SMMs behaviour after anchoring on the surface of silica nanoparticles. However, the shape of *M(H)* curves do not show, step-like features as described in literature for the bulk Mn${}_{12}$-st [11,16,19]. On the other hand, typical butterfly shape, which points to quantum tunnelling of magnetization (QTM), were recognized for all the concentrations [20]. Here, the QTM is probably influenced by some distortion of magnetic molecules ligand structure upon grafting [21] or changes in the magnetic anisotropy with respect to the bulk phase [22].

Interesting is that both coercive field and remanence magnetization did not show monotonic dependence on the number of spacers per single anchoring unit. The samples SilS-Mn${}_{12}$N1 and SilS-Mn${}_{12}$N3 show similar characteristics with a coercivity of 1.32 kOe and 1.16 kOe and remanence of 0.029 emu/g and 0.023 emu/g, respectively. This is consistent with the fact that both samples contain the same concentration of SMMs and the difference lays in the number of attaching units [13]. The width of the hysteresis loop is maximum for the SilS-Mn${}_{12}$N6 sample and reveals $H_{c}$ = 4.51 kOe and $M_{rem}$ = 0.072 emu/g. Increase of coercivity and remanence of such a sample, in contrast to other, can be explained looking on the structural and morphological analysis. At the disk-like core of Mn${}_{12}$-st, eight organic ligands are located above and below the largest molecular plane, along the easy axis, and other eight ligands are located equatorially on the circumference of the molecule, perpendicular to the easy axis (see: Figure 3a). Preferably Mn${}_{12}$-st molecules are attached to the surface via propyl-carbonic acid link in an umbrella-like arrangement when the largest surface plane of the SMMs is perpendicular to the silica surface and have from two to four bonding with the substrate [12]. However, as it was shown above, the concentration of six spacer units for one functional group involves the distortion from such orientation and makes it possible to deposit some of the molecules by the single bond, which lead to significantly greater freedom of movement of Mn${}_{12}$ molecules. It is known that the molecular orientation on the surface is one of the key ingredients of the magnetic behaviour of SMMs [23] and can directly affect the width of the hysteresis loop. We can, therefore, expect, that such single-bond deposition of SMMs in SilS-Mn${}_{12}$N6 sample allows some individual molecules to have a preferential orientation in the external magnetic field and align easy axis magnetization along the external field. The SilS-Mn${}_{12}$N9 sample, similar to sample SilS-Mn${}_{12}$N6, retains the orientation freedom of certain molecules, however the number of anchored Mn${}_{12}$-st is lower, and the coercive field reduces to $H_{c}$ = 2.55 kOe ($M_{rem}$ = 0.047 emu/g).

The high field results of the isothermal magnetization measurements cause some difficulties in interpretation. All the samples do not reveal saturation of magnetization as it was expected for Mn${}_{12}$-st SMMs in the powder state. Therefore, the *M* vs. *H* data cannot be used to qualitatively calculate the amount of deposited Mn${}_{12}$ molecules. However, the increase of magnetization for SilS-Mn${}_{12}$N6 is significantly higher than for other samples. This stays in agreement with our previous research [13], in which the DPASV measurements confirmed that SilS-Mn${}_{12}$N6 possess the largest possible numbers of immobilized SMMs with the smallest possible numbers of bonds. In other words SilS-Mn${}_{12}$N6 have the largest number of Mn${}_{12}$ molecules with freedom of movement, which helps to align easy axis magnetization along the external field.

**Figure 8:**
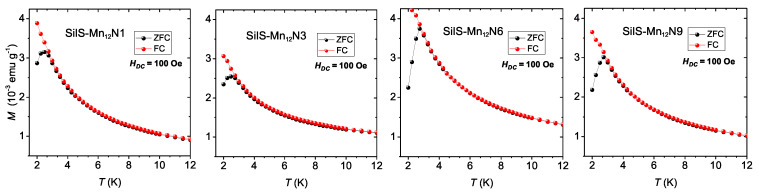
Magnetization dependence on the temperature for value of magnetic field *H* = 100 Oe of SilS-Mn${}_{12}$NX (X = 1, 3, 6, 9) samples.

The magnetization as a function of temperature for samples at an external field of 100 Oe is presented in Figure 8. The bifurcation of zero-field cooled (ZFC) and field-cooled (FC) magnetization curves indicate the blocking temperature, $T_{B}$, below which magnetic hysteresis of the samples occurs. All the samples show similar blocking temperature of about 2.7 K, which is comparable with earlier reported blocking temperature (∼3.0 K) for bulk crystalline Mn${}_{12}$-st [16,19] and higher than reported for analogous Mn${}_{12}$-stearate-based, honeycomb-patterned films (∼2.0 K) [24].

**Figure 9:**
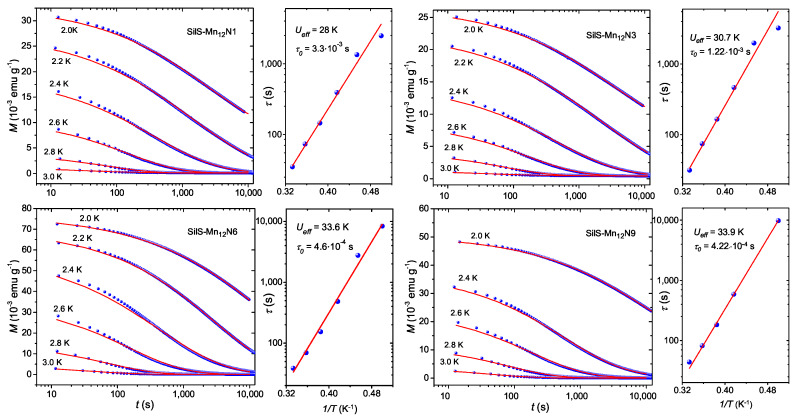
Dependence of magnetization on the time for SilS-Mn${}_{12}$NX (X = 1, 3, 6, 9) samples at the 2.0–3.0 K temperature range (the solid lines are the best fits to the stretched exponential function (1)) and corresponding relaxation times in function of inverse temperature (solid lines represent the best fit to the Arrhenius function (2)).

The magnetic relaxation measurements, performed for the temperature range 2.0 K–3.0 K, are presented in Figure 9. The magnetization values at 2.0 K of the first recorded point reveal that, SilS-Mn${}_{12}$N1 and SilS-Mn${}_{12}$N3 preserves about 20% of the value measured at 50 kOe. In case of SilS-Mn${}_{12}$N6 and SilS-Mn${}_{12}$N9 samples, this factor is higher and reaches about 30% of the value measured at 50 kOe. To qualitatively evaluate the magnetic relaxation, the time dependence of the magnetization data were analyzed with the stretched exponential function [25]:

(1) $$M\left(t\right)=\sigma_{0}+M_{0}\exp{\left(-t/\tau \right)}^{\beta },$$

where $M_{0}$ is an initial magnetization value, τ is the mean relaxation time, β (0 < β < 1) describes the distribution of the relaxation time and $\sigma_{0}$ is an offset parameter. The offset parameter is close to zero for all measured temperatures as it was expected for such a magnetic compound. The obtained β parameters are temperature-dependent reaching 0.40–0.43 values at 2.0 K and 0.58–0.63 at 2.8 K. The linear increase of β parameter with temperature is in agreement with previous reports for bulk Mn${}_{12}$-st [16,19] and other derivate compounds of Mn${}_{12}$-acetate [25,26,27]. However, the β values for higher temperatures are slightly lower than expected. One of the possible explanation could be the structure distortion of Mn${}_{12}$ after deposition, which affects the distribution of the transverse anisotropies responsible for stretched exponential behaviour of *M* vs. time [22]. Let us mention that temperature dependence of relaxation rate may indicate that Quantum Tunneling of Magnetization effect (QTM) is thermally assisted for all samples [16].

The temperature dependence of obtained relaxation times (Figure 9) can be fitted to the Arrhenius law to estimate the energy barrier $U_{eff}$ for the reorientation of magnetization:

(2) $$\tau \left(T\right)=\tau_{0}\exp\left(U_{eff}/k_{B}T\right),$$

where the pre-exponential factor $\tau_{0}$ is the relaxation time in the high-temperature limit and $k_{B}$ is Boltzmann constant. The obtained parameters for all investigated samples are summarized in the Table 1. Fits to the above equation show small differences of parameters between samples with different number of spacers per single anchoring unit. The SilS-Mn${}_{12}$N1 and SilS-Mn${}_{12}$N3 samples reveal reduced barrier values in compare to other two samples and show small deviation from linear behavior on semi-logarithmic plot of the relaxation time. The values of $U_{eff}$ and $\tau_{0}$ for SilS-Mn${}_{12}$N6 and SilS-Mn${}_{12}$N9 samples are $U_{eff}$ = 33.6 K, $\tau_{0}$ = 4.6 × 10${}^{-4}$ s and $U_{eff}$ = 33.9 K, $\tau_{0}$ = 4.22 × 10${}^{-4}$ s, respectively, which is close to the earlier reported result [16] for analogous bulk compound ( $U_{eff}$∼ 40 K and $\tau_{0}$ = 1.03 × 10${}^{-4}$ s).

**Figure 10:**
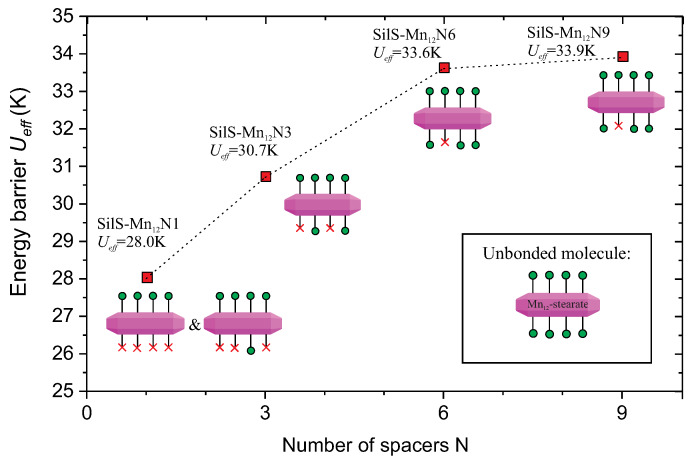
Dependence of energy barrier $U_{eff}$ on the number of spacer units in the sample. Below the points the graphical illustration of the anchored molecules can be seen. The green circles means full stearate ligand, while red crosses represent the anchoring points.

Nevertheless, looking at the overall trend (Figure 10), we can notice that the energy barrier $U_{eff}$ is slowly, but continuously increasing, with the increasing of the number of spacer units to the six spacers (SilS-Mn${}_{12}$N6). The SilS-Mn${}_{12}$N9 sample presents almost the same energy barrier, as the SilS-Mn${}_{12}$N6 one. Both samples (SilS-Mn${}_{12}$N6 and SilS-Mn${}_{12}$N9) have SMMs anchored in a similar way by a single bonding point, while for samples SilS-Mn${}_{12}$N1 and SilS-Mn${}_{12}$N3 deposited SMMs have more anchoring points. Thus, the value of energy barrier can be related with the structure of the anchored Mn${}_{12}$-st molecules. The ideal Mn${}_{12}$-st molecule possesses 16 stearate acid ligands: eight at the circumference, four at the upper side and next four at the bottom. Attaching the molecule to the silica’s surface with a single point involves substitution of a single stearate unit with a propyl carbonate one, double point involves two substitutions and so on up to four [13]. Assuming the umbrella-like configuration of the Mn${}_{12}$-st, the SilS-Mn${}_{12}$N1 sample has the most distorted and anisotropic structure, SilS-Mn${}_{12}$N3 is less distorted, while SilS-Mn${}_{12}$N6 and SilS-Mn${}_{12}$N9 materials have the least perturbed structures and the most symmetrical (both samples presents a similar way of the anchoring of SMMs). Therefore, the observed differences in the energy barrier can be explained as a consequence of the deposition on the surface, which introduce the modification of axial anisotropy. In consequence, these leads to the modification of the energy barrier for deposited SMMs in contrast to the bulk ones [28,29,30].

As it was shown, Mn${}_{12}$-st SMMs can be successfully deposited on the surface of the spherical silica and their magnetic properties can be tuned by the number of spacers per single anchoring unit. The presence of long chains of stearate ligands make such molecule magnets a great candidate for surface deposition, preventing its inner structure from degrading and providing molecules stability. Also important is the fact that the slow relaxation process is retained for molecules with different number of spacers per single anchoring unit. This is additional confirmation that SMMs have a molecular origin of magnetization and hysteresis appears as the property of the isolated molecule and not due to a collective behaviour [31,32,33,34].

### 3.3. Conclusions

In summary, we studied the magnetic behaviour of Mn${}_{12}$-st single-molecule magnets anchored to the surface of the silica substrate with different concentration of spacer units. The successful functionalization of the surface by SMMs was confirmed by TEM microscopy and Raman vibrational analysis, which revealed the way of SMMs immobilization and the structure of the samples. The series of static and dynamic magnetic measurements were used for characterization of molecular magnetic properties of SMMs organized on the surface. By assuming the different mobility (degrees of the freedom) of SMMs and different concentration of spacer units, all of the samples have shown preservation of hysteretic magnetic behaviour and slow relaxation properties, characteristic for such Mn${}_{12}$ complex. The most promising sample is determined to be SilS-Mn${}_{12}$N6, possessing six spacer units per one anchoring and having the possibility of preferential orientation of some deposited SMMs in the magnetic field. For such a sample, the most coercive field and remanence magnetization were observed. Also, SilS-Mn${}_{12}$N6 and SilS-Mn${}_{12}$N9 samples show slow relaxation in time-dependent magnetization with the value of energy barrier close to expected for such SMMs. Observed slight changes of magnetic properties in comparison to a bulk structure can be contributed to the possible modifications of SMMs anisotropy upon the grafting. Performed structural and detailed magnetic analysis confirms the possibility of functionalization of the surface by Mn${}_{12}$-st SMMs with preservation of typical magnetic behaviour which opens a possibility for various applications of such materials in nanoelectronics.

## Figures and Tables

**Table 1:** Static and dynamic magnetic characteristics for SilS-Mn${}_{12}$ NX (X = 1, 3, 6, 9) samples.

Sample	$H_{c}$ (kOe)	$M_{\mathrm{rem}}$ (emu g${}^{-1}$)	$T_{B}$ (K)	$U_{\mathrm{eff}}$ (K)	$\tau_{0}$ (s)
SilS-Mn${}_{12}$N1	1.32	0.029	2.65	28 ± 1.5	3.3 × 10${}^{-3}$
SilS-Mn${}_{12}$N3	1.16	0.023	2.65	31 ± 2	1.1 × 10${}^{-3}$
SilS-Mn${}_{12}$N6	4.51	0.072	2.74	33.6 ± 1.7	4.6 × 10${}^{-4}$
SilS-Mn${}_{12}$N9	2.55	0.047	2.74	33.9 ± 1.6	4.2 × 10${}^{-4}$

## Abbreviations

The following abbreviations are used in this manuscript:

Mn${}_{12}$ac${}_{16}$	$\left[{\mathrm{Mn}}_{12}O_{12}{\left({\mathrm{CH}}_{3}{\mathrm{CO}}_{2}\right)}_{16}\right]\cdot 2{\mathrm{CH}}_{3}\mathrm{COOH}\cdot 4H_{2}O$
Mn${}_{12}$-st	derivative of Mn${}_{12}$ containing strearic acid ligands: [${\mathrm{Mn}}_{12}O_{12}{\left({\mathrm{CH}}_{3}{\left({\mathrm{CH}}_{2}\right)}_{16}{\mathrm{CO}}_{2}\right)}_{16}]\cdot 2{\mathrm{CH}}_{3}\mathrm{COOH}\cdot 4H_{2}O$
TEOS	tetraethyl orthosilicate
BNTES	butyronitriletriethoxysilane
ClTMS	chlorotrimethylsilane
Me	methyl groups
Et	ethyl units
QTM	quantum tunnelling of magnetization
